# Isolating the Effects of Word’s Emotional Valence on Subsequent Morphosyntactic Processing: An Event-Related Brain Potentials Study

**DOI:** 10.3389/fpsyg.2018.02291

**Published:** 2018-11-21

**Authors:** Javier Espuny, Laura Jiménez-Ortega, David Hernández-Gutiérrez, Francisco Muñoz, Sabela Fondevila, Pilar Casado, Manuel Martín-Loeches

**Affiliations:** ^1^Cognitive Neuroscience Section, UCM-ISCIII Center for Human Evolution and Behavior, Complutense University of Madrid, Madrid, Spain; ^2^Department of Psychobiology & Behavioral Sciences Methods, Complutense University of Madrid, Madrid, Spain

**Keywords:** language, morphosyntactic processing, emotion, valence, arousal, ERP, LAN, P600

## Abstract

Emotional information significantly affects cognitive processes, as proved by research in the past decades. Recently, emotional effects on language comprehension and, particularly, syntactic processing, have been reported. However, more research is needed, as this is yet very scarce. The present paper focuses on the effects of emotion-laden linguistic material (words) on subsequent morphosyntactic processing, by using Event-Related brain Potentials (ERP). The main aim of this paper is to clarify whether the effects previously reported remain when positive, negative and neutral stimuli are equated in arousal levels and whether they remain long-lasting. In addition, we aimed at testing whether these effects vary as a function of the task performed with the emotion-laden words, to assess their robustness across variations in attention and cognitive load during the processing of the emotional words. In this regard, two different tasks were performed: a reading aloud (RA) task, where participants simply read aloud the words, written in black on white background, and an Emotional Stroop (ES) task, where participants named the colors in which the emotional words were shown. After these words, neutral sentences followed, that had to be evaluated for grammaticality while recording ERPs (50% containing a morphosyntactic anomaly). ERP analyses showed main effects of valence across tasks on the two components reflecting morphosyntactic processing: The Left Anterior Negativity (LAN) is increased by previous emotional words (more by negative than positive) relative to neutral ones, while the P600 is similarly decreased. No interactions between task and valence were found. As a result, an emotion-laden word preceding a sentence can modulate the syntactic processing of the latter, independently of the arousal and processing conditions of the emotional word.

## Introduction

Contrary to previous traditions in the cognitive sciences, emotions seem to strongly impact cognitive processes. Numerous experiments have demonstrated how positive or negative emotions affect processes such as perception, attention, memory, creativity and other higher cognitive functions (see, for instance, Ashby et al., 1999; Mitchell and Phillips, 2007; Pessoa, 2008).

Several experiments explored how emotional response mechanisms compete for resources with non-emotional cognitive processes, as shown by an increase of the brain activity in right frontal areas versus neutral stimuli (Gupta and Raymond, 2012; and so do motivational mechanisms: Gupta et al., 2018). Further, the time course of visual attention would depend on the emotional content (Srivastava and Srinivasan, 2010), suggesting that positive stimuli are more related to broad scope attention, which needs less attentional resources (Srinivasan and Gupta, 2011), than negative stimuli, more related to focused attention (Srinivasan and Gupta, 2010; Srinivasan and Hanif, 2010; Srivastava and Srinivasan, 2010; Gupta and Srinivasan, 2015; Gupta et al., 2016).

Thus, evidence supports that emotional information influences subsequent non-emotional tasks, including language processing (e.g., Gupta and Raymond, 2012) and, particularly, sentence-morphosyntactic processing (e.g., Jiménez-Ortega et al., 2012, 2017; Verhees et al., 2015). This has been a highly relevant finding, given that syntax has been traditionally described as automatic, modular and encapsulated, blind to other processes (Hauser et al., 2002; Friederici, 2004).

Most of the straightforward evidence on the influence of emotions on syntactic processing comes from Event-Related brain Potentials (ERP). In this regard, emotional information has been able to modulate at least two ERP components normally related to morphosyntactic processing. One of these components is the Left Anterior Negativity (LAN), triggered by syntactic anomalies (Bohn et al., 2013; Magne et al., 2016). LAN is supposed to reflect the detection of syntactic anomalies, difficulty of processing of rare structures, or incapacity of matching a word within a phrase structure (Hahne and Friederici, 1999; Hagoort, 2003), as well as some aspects of working memory processes (Coulson et al., 1998; Martín-Loeches et al., 2005), and represents the difference between syntactically incorrect and correct sentences. Another component typically related to morphosyntactic processing is the late P600 component, a positive parietal deflection considered to reflect the integration of the current information with previous contextual data, being also related to structural mismatches –as disagreement– or non-preferred continuations of the sentence (Friederici et al., 2002; Martín-Loeches et al., 2009), also representing the difference between syntactically incorrect and correct sentences. However, its exact nature is being matter of intense debate (e.g., Sassenhagen and Bornkessel-Schlesewsky, 2015).

Some studies have explored the impact of emotional information on morphosyntactic processing when emotion is enclosed within the sentence to be syntactically analyzed (short-term effects) (Martín-Loeches et al., 2012; Jiménez-Ortega et al., 2017). Overall, these studies have reported differential effects as a function of valence, and the results have been related to divergent cognitive strategies induced by emotions during morphosyntactic processing. Other approaches to the effects of emotions on syntactic processing have presented emotion-laden information before the sentence to be processed (long-term effects). In Jiménez-Ortega et al. (2012), emotion-laden paragraphs (positive, negative, and versus neutral) preceded neutral sentences that could be morphosyntactically correct or incorrect. The LAN component was not visible in the neutral condition, but it was triggered in negative and positive conditions. The results suggested modulatory effects of prior emotions on syntactic processing. One explanation would be a boosting of the LAN by arousal modulations, since the LAN component was similar for positive and negative conditions. However, arousal levels of paragraphs across valences was not equated, so there is no evidence disentangling the real factor (valence or arousal) underlying these long-term emotional effects on morphosyntactic processing. On the other hand, using happy and sad (no neutral condition was included) film clips preceding sentences containing subject-verb agreement violations, some studies have shown modulations of the P600 component (Vissers et al., 2010; Verhees et al., 2015). Results showed a reduction of the P600 component for sad mood compared to happy mood. Furthermore, Verhees et al. (2015) drove participant’s attention either to the syntactic task or to a physical (letter size) one. The P600 to incorrect sentences was affected by the task performed. When the attention was directed to syntactic processing, positive induction increased the P600, while negative mood decreased it: in the size task, positive induction reduced the P600.

Valence has not been manipulated independently from arousal in the previous studies aiming to study the impact of emotional information on syntactic processing, yet specific experiments are required in this regard, matching arousal degree between valence conditions, including (importantly) the neutral one. The goal of the present study is to contribute in this respect. The long-term effects of valence on morphosyntactic processing irrespective of arousal are here explored. For the valence manipulation, positive, negative, and neutral words were presented prior to neutral sentences with or without morphosyntactic violations, as did Jiménez-Ortega et al. (2012) with whole paragraphs. Since it is not possible to find highly arisen neutral words (see Lang et al., 1998; Bradley and Lang, 1999), the arousal values of all three emotional conditions were matched to a medium value. In a recent study, Espuny et al. (2018) used the same affective words equated in arousal to explore valence-related ERP fluctuations. The valence of the words resulted in differential effects on Early Posterior Negativity (EPN) and Late Positive Complex (LPC) components of negative versus positive words (reflecting a negativity bias). With these previous findings, we aim to explore whether the same differential effects of valence persist in the long-term, that is, in the syntactic processing of a neutral sentence subsequent to emotion-laden words’ presentation, by differentially modulating the LAN and/or the P600 components.

Espuny et al. (2018) also explored whether the emotional stimuli (affective words) were differentially processed when their meaning was fully accessed or not (automatic semantic access versus diminished semantic access). In this regard, participants performed two types of tasks, when the valenced words were presented: an effortless task (baseline) simply consisted in reading aloud (RA) task a word written in black on white, in a natural reading situation, that implies an automatic activation of the semantic network. The other task was an emotional Stroop (ES) task, consisted in RA the color (out of 6 possible) in which the word was presented. This task involves processes turning word processing more effortful and problematic, such as cognitive load and interference effect, then biasing the attentional resources to the emotional information (Dresler et al., 2009), presumably weakening semantic word process. The task has also been probed to enhance arousal (Teixeira-Silva et al., 2004). In Espuny et al. (2018), no effects of task were found in word processing. However, in the present experiment, we maintain these different tasks in the emotional words’ presentation preceding the neutral sentence to check whether even poorly attended emotional information could affect syntactic processing in the long-term (around 2 s). Some studies have reported the effects on morphosyntactic processing of emotional words presented subliminally within the sentence; the present study extends this idea by presenting poorly attended words (meaning is inhibited) preceding the sentence.

In view of previous findings, meanly those by Espuny et al. (2018) in which the emotion-laden material used here yielded differential effects as a function of valence, we expect a dissociated valence effects on subsequent sentence processing, while arousal is being controlled. This should be reflected in different modulations of the LAN and P600 ERP components as a function of the sign (positive or negative) of the valence. If the long-term effects of emotion on sentence processing are robust enough as to take place even when the emotion-laden stimulus has been partly of defectively attended, we also expect dissociated modulations as a function of the task performed during the presentation of the valenced words.

## Materials and Methods

### Participants

Twenty-four native Spanish speakers (17 women; mean age = 25.5 years; and *SD* = 8.37) participated in this experiment. All of them were university students and gave informed consent before the experiment. The study was performed in accordance with the Declaration of Helsinki and approved by the ethics committee of the Complutense University, Madrid, Spain. All the participants reported normal or corrected-to-normal vision and no history of neural or cognitive disorders or reading difficulty. All were right-handed, ranging from 30 to 100 (*M* = 83) according to the Edinburgh Handedness Inventory (Oldfield, 1971). After the experiment they filled-in a STAI questionnaire (State-Trait Anxiety inventory) (Spielberger, 1983) to measure whether any of them had an out-of-normal state or trait anxiety. The results were normal, with a state-anxiety mean of 71.5 (*SD* = 9.8) and a trait-anxiety mean of 66.6 (*SD* = 15.3).

### Materials

We constructed 360 Spanish sentences of neutral valence. The structure of the sentences [(determiner)-(noun)-(adjective)-(verb)] was like other sentences already used in previous experiments (Jiménez-Ortega et al., 2012, 2014; Martín-Loeches et al., 2012). Half of the sentences were constructed in singular, the other half in plural. Since Spanish has two grammatical genders, half of the sentences were constructed with masculine words and the other half with feminine ones.

Our critical word (CW) in the sentences was the adjective. We selected neutral adjectives from the Spanish adaptation of the ANEW (Redondo et al., 2007). Adjectives’ mean valence was 5.1 (*SD* = 0.73) and arousal mean was 4.7 (*SD* = 0.8) in a 1–9 scale. Accordingly, they could be considered of neutral valence and medium arousal. For each sentence, two additional ones were generated either containing a gender disagreement or a number disagreement between noun and adjective. That is, we had 360 correct sentences, 360 sentences with a gender disagreement in the adjective and 360 sentences with a number disagreement in the adjective. Examples of the sentences can be found in Table 1.

**Table 1:** Examples of experimental sentences and their English literal translation.

Correct sentences	(a) El Ganado **lanudo** pasta {The cattle [masc., sing.] **shaggy** [masc., sing.] grazes} (b) El detective **privado** investiga {The detective [masc., sing.] **private** [masc., sing.] investigates}
Gender disagreement	(a) El Ganado **lanuda** pasta {The cattle [masc., sing.] **shaggy** [fem., sing.] grazes} (b) El detective **privada** investiga {The detective [masc., sing.] **private** [fem., sing.] investigates}
Number disagreement	(a) El Ganado **lanudos** pasta {The cattle [masc., sing.] **shaggy** [masc., plur.] grazes} (b) El detective **privados** investiga {The detective [masc., sing.] **private** [masc., plur.] investigates}

*Critical words are in bold.*

To counterbalance stimuli presentation, the complete scenario of 360 sentences had to be presented to each participant in six sets of 60 sentences each. Every set was constructed with 30 correct and 30 incorrect sentences (half of the incorrections consisted on gender disagreements, half on number disagreements). Within every set, the 60 sentences were randomly mixed during the presentation. A total of 12 different combinations of 6 sets each were constructed, so that each participant viewed every one of the 360 sentences only once and in only one version (correct or one of the two incorrect versions). Across the 12 combinations, all the sentences appear counterbalanced across versions, in different participants. Since we had 12 combinations and 24 participants, each combination was presented twice.

In addition, a list of 180 emotional words was constructed, to be presented preceding each experimental sentence. These words were those used in Espuny et al. (2018). Each emotional word appeared twice in two different tasks (ES vs. RA). Words were selected considering the activation and valence values provided by the Spanish adaptation of ANEW (Redondo et al., 2007). Sixty words were selected (30 nouns, 30 adjectives) for each valence condition: negative, neutral and positive. The average valence for positive words was 7.46 (*SD* = 0.36), for negative words was 2.29 (*SD* = 0.48), and for neutral ones was 5.01 (*SD* = 0.56), in a 1–9 scale. Arousal levels were carefully matched across conditions to such an extent that the mean arousal value for positive, negative and neutral condition was the same, 5.03, with *SD* = 0.53, *SD* = 0.55, and *SD* = 0.52, for positive, negative, and neutral condition, respectively.

### Procedure

The experiment was block-designed. The use of the emotional words as mood inductors before the sentences resulted in six different conditions: positive, negative and neutral valence, each one both in two tasks —ES and RA. Every block consisted of 60 emotional words (30 nouns, 30 adjectives), each preceding one of the 60 sentences in a set (see above). All the emotional words in a block had the same valence, to best maintain the induced mood. So, each one of the six blocks had a single combination of valence and task.

The RA task consisted in RA the single word presented in black on white prior to the sentence. In the ES task, participants had to inhibit the meaning of the word and read aloud the color in which it was presented. Six different colors were used: red, green, blue, yellow, magenta, and orange. In every block of 60 words, we used 10 times each color, randomizing the order.

**FIGURE 1:**
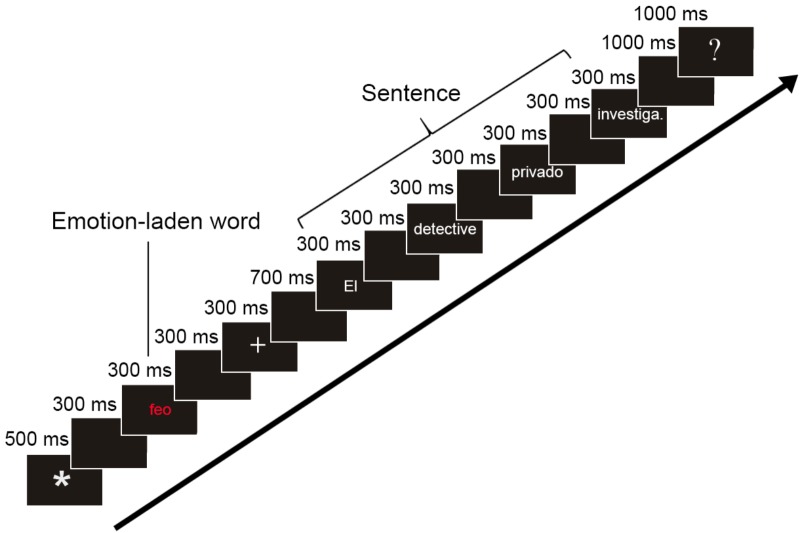
Stimulation procedure. Schematic representation of procedure trial: emotional induction (emotion-laden word) is followed by a neutral sentence, in which the adjective could be morphosyntactically agreeing or disagreeing with the preceding noun. The translation into English of the example is “ugly” for the emotion-laden word and “The private detective investigates” for the subsequent sentence.

Participants were comfortably seated in a quiet shielded chamber watching an LCD screen (placed 65 cm from their eyes, visual angles around 0.8–4° width). Each trial began with an asterisk, lasting 500 ms. After an inter-stimulus-interval (ISI) of 300 ms, the emotional stimulus (adjective or noun, in black on white in the RA task; in color against white in the ES task) was presented during 300 ms. Other 300 ms of blank were followed by a fixation cross for 500 ms, indicating the sentence was about to appear. After 700 ms of blank screen, the sentence started, word by word, in black on white, in the center of the monitor, lasting 300 ms each word and with 300 ms of ISI. At the end of the sentence, after 1 s, a question mark was presented for 1 s, lending the subject to respond whether the sentence was correct or incorrect. The first word of each sentence always began with a capital letter; the last word was always presented with an ending dot. The used font was 30-point Arial (Figure 1). A keypad was used with two buttons to respond about the correctness of the sentence with index and middle fingers. The response hand was counterbalanced.

Before the experimental trials, the subjects were instructed about the experimental procedure. After a 6-trial training to ensure that subjects understood the procedure, the experiment started. The first three blocks of 60 trials (i.e., emotional word followed by a sentence) used always the same task, that is, either RA or ES. Between blocks, a message of pause appeared; subjects had to press a button to continue. Between the third and the fourth block, preceding task changed (from RA to ES task, or vice versa, this counterbalanced). Since the level of arousal was expected to vary between the two parts of the experiment, enough time between the 3rd and the 4th blocks was necessary (especially if the session started with the ES task) to return to the arousal baseline values. The Oldfield test of handedness was filled-in at this moment. Then, when participants were ready, instructions about the new preceding task were given, followed by a training period, before starting with the next –and last- three blocks, with pauses between them. Finally, the participants completed the STAI questionnaire.

### Skin Conductance Recording

Skin conductance (SC) was recorded throughout the experiment by means of a Biofeedback (6-channel Psymtec I-330-C2), connected to the index and middle fingers of the hand that was not used to respond. This manner, we gathered information regarding the arousal levels of the participant, to contrast it across conditions. We compared SC means between conditions, after normalizing data within subjects: all individual measures of SCR in μS were averaged for each participant separately. This grand mean was assigned a value of one; thereafter, the specific values as a function of each preceding task were obtained and compared in terms of standard deviations from the grand mean.

### Electroencephalographic Recording

The electroencephalogram (EEG) was recorded with 59 electrodes attached in a cap (Electro Cap International) with the locations: Fp1, Fpz, Fp2, AF7, AF3, AF4, AF8, F7, F5, F3, F1, Fz, F2, F4, F6, F8, FT7, FC5, FC3, FC1, FCz, FC2, FC4, FC6, FT8, T7, C5, C3, C1, Cz, C2, C4, C6, T8, TP7, CP5, CP3, CP1, CPz, CP2, CP4, CP6, TP8, P7, P5, P3, P1, Pz, P2, P4, P6, P8, PO7, PO3, PO4, PO8, O1, Oz, and O2, plus the right mastoid (M2). All of them were initially referenced to the left mastoid (M1). For controlling ocular movements, EOG were registered with electrodes above and below the left eye (VEOG), and at the outer canthus of each eye (HEOG), for off-line eye-movement correction. A Brainamp amplifier (Brain Products, GmbH) was used, keeping electrode impedances below three kΩ. The signal was continuously recorded with a bandpass from 0.01 to 30 Hz and a sampling rate of 250 Hz.

### Data Analysis

The data analysis was performed using Brain Vision Analyzer software (Brain Products, GmbH). Continuous recording of EEG was divided into epochs of 1000 ms each, starting 200 ms before the onset of the CW. We considered only correct trials, discarding incorrect and out-of- time trials. We corrected eye blinks and EOG artifacts using a semiautomatic Ocular Correction ICA tool (fast-ICA extended); final components rejection was done by visual inspection. Similarly, other artifacts rejection was semiautomatic: an automatic rejection of epochs exceeding ±100 μV was followed by a manual rejection by visual inspection. Data of the 59 locations was referenced again by calculating the mean between M1 and M2 data.

**FIGURE 2:**
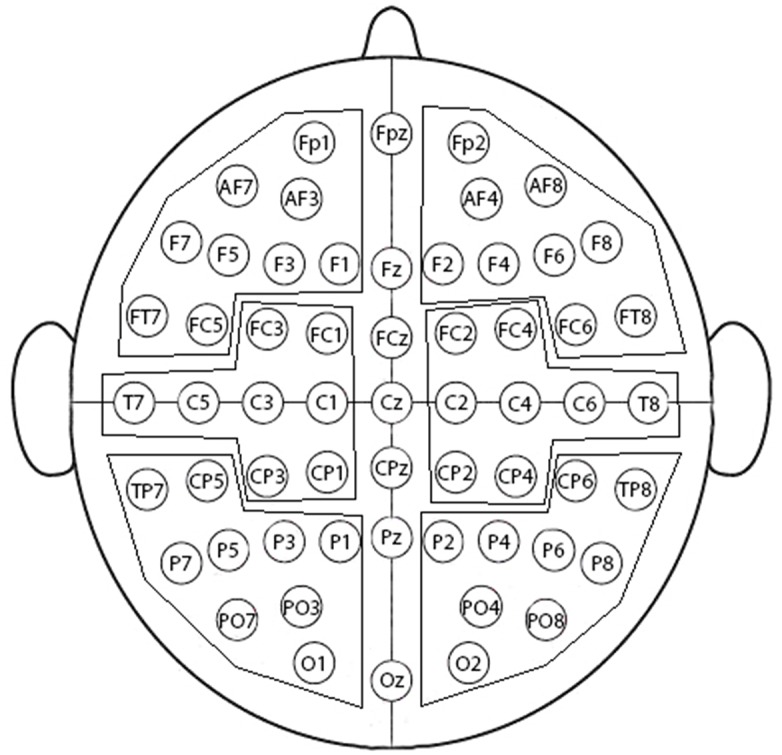
Brain Map. Topographic brain map of the scalp electrodes grouped in regions of interest (ROI) chosen for analysis: left anterior, right anterior, left central, right central, left posterior, and right posterior regions.

Statistical analyses of variance (ANOVAs) of ERP data were performed using six brain regions of interest (ROIs): anterior, central and posterior regions, each one represented in the two hemispheres (Figure 2). The factors in the ANOVAs were: Region (anterior, central, and posterior), Hemisphere (left, right), Correctness (morphosyntactically correct, incorrect), Preceding task (RA, ES) and Valence (positive, negative, and neutral). The violations of the sphericity assumption were corrected when necessary by the Greenhouse-Geiser method, and *post hoc* tests were corrected by the Bonferroni procedure. Specific time windows for the analyses were selected based upon visual inspection of the main ERP components.

## Results

### Behavioral Data

Participants appropriately judged the morphosyntactically incorrect sentences more accurately [93.3% (*SD* = 4.7)] than the correct ones [91.8% of cases (*SD* = 5.2)]. This difference was significant, as supported by a main effect of Correctness [*F*(1,23) = 6; *p* = 0.023; η2 = 0.214; and π = 0.649] in an ANOVA comprising Correctness, Preceding task and Valence. There were no significant differences regarding other factors or interactions. [all *F*s < 0.58, *p* > 0.1].

In line with the error rates findings, reaction times were faster (mean 427 ms, *SD* = 135 ms) for incorrect sentences than for correct ones (mean 443 ms, SD = 132 ms), showing a main effect [*F*(1,22) = 7.74; *p* = 0.011; η2 = 0.26; and π = 0.758].

Trials judged as morphosyntactically correct when they were incorrect or vice versa were discarded in further ERP analyses.

### Skin Conductance

The comparison showed a main effect of Preceding task [*F*(1,23) = 4.78; *p* = 0.039; η2 = 0.172; and π = 0.554]. As expected, ES task produced a significant increase of SC (standardized *M* = 1.03) compared to the RA task (standardized *M* = 0.97). No effects were found on SC concerning valence conditions [*F*(2,46) = 2.61; *p* = 0.088; η2 = 0.102; and π = 0.479].

### Event-Related Potentials

#### LAN

**FIGURE 3:**
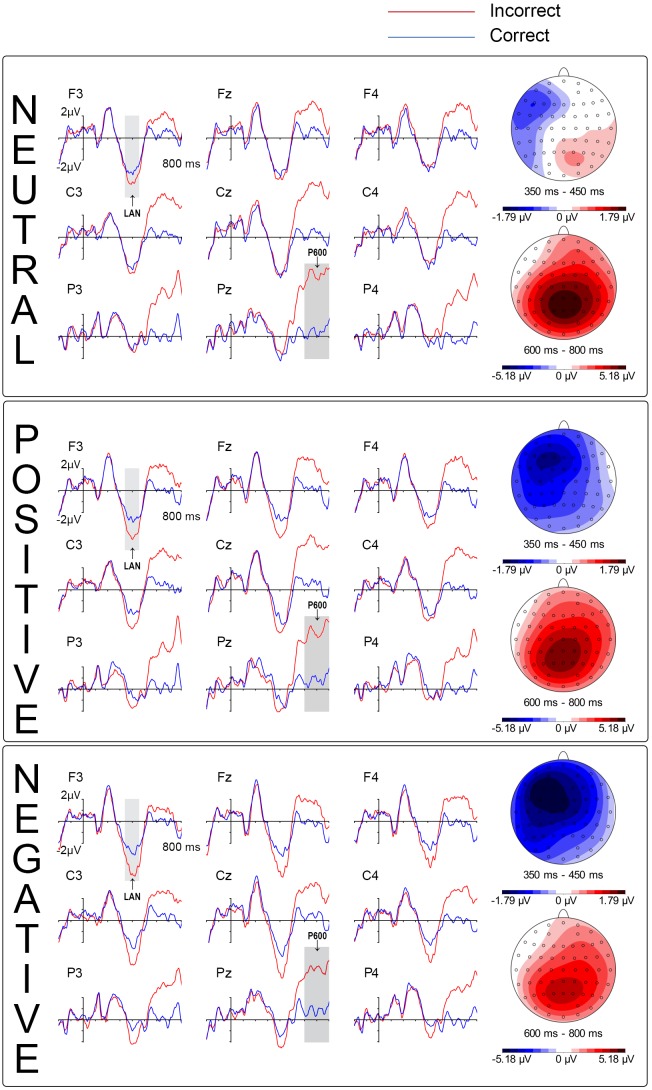
Left Anterior Negativity (LAN) and P600. Event-Related brain Potential (ERP) of the Correctness by Valence interactions: LAN, and P600.

Visual inspections revealed a LAN for the three valence conditions between 350 and 450 ms, with different wave amplitudes, being largest in the negative valence, slightly smaller in the positive, and showing the lowest values in the neutral condition (Figure 3). In the 350–450 ms time window, a significant effect of Correctness appeared [*F*(1,23) = 13.39; *p* = 0.001; η2 = 0.368; and π = 0.939], as well as of a Correctness x Valence interaction [*F*(2,46) = 4.3; *p* = 0.021; η2 = 0.158; and π = 0.71]. Preceding Task x Region yielded significant effects [*F*(2,46) = 5.04; *p* = 0.017; η2 = 0.18; and π = 0.722] while the Preceding Task x Correction x Hemisphere interaction resulted in a trend [*F*(1,23) = 4.03; p = 0.056; η2 = 0.149; and π = 0.486]. All other factors and interactions were not significant [all *F*s < 1.65, *p* always >0.1]. *Post hoc* analyses revealed that the LAN differed significantly between neutral and negative conditions [*F*(1,23) = 11.74; *p* = 0.033; η2 = 0.248; and π = 0.751]. No significant differences appeared between neutral and positive conditions [*F*(1,23) = 4.05; *p* > 0.1; η2 = 0.15; and π = 0.488] neither between the positive and negative conditions in the LAN [*F*(1,23) = 0.87; *p* > 0.1; η2 = 0.036; and π = 0.146]. Statistically, negative and positive conditions behave similarly, but also positive and neutral conditions, therefore an intermediate position of positive condition between neutral and negative conditions might be suggested, as a visual inspection of the waves illustrates. Preceding Task effects will not be discussed, because of two main reasons: first, the effects did not interact with Correctness, so they are not affecting the syntactic processes of interest here. Second, there is no straightforward evidence on the specific factor underlying the effect, since different number of them could be implicated (i.e., arousal level, cognitive load, interference effect, and attentional bias).

### P600

Visual inspection revealed a P600 mainly between 600 and 800 ms, apparently modulated by valence. ANOVAs in the 600–800 ms window yielded significant effects of Correctness [*F*(1,23) = 40.339; *p <* 0.001; η2 = 0.637; and π = 1], and a trend to significance of Correctness x Valence x Region [*F*(2,46) = 2.38; *p* = 0.092; η2 = 0.094; and π = 0.507]. Since the P600 is distributed mainly in the central and posterior regions, and in order to seemingly reduce statistical power issues, a new ANOVA excluding the anterior region was performed, yielding a main effect of Correctness [*F*(1,23) = 50.19; *p* < 0.001; η2 = 0.686; and π = 1] and of Correctness x Valence [*F*(2,46) = 3.22; *p* = 0.050; η2 = 0.123; and π = 0.579]. *Post hoc* analyses comparing the P600 amplitude for each valence (subtracting the activation of the correct ones to the incorrect ones) revealed a significant difference between negative and neutral conditions [*F*(1,23) = 7.54; *p* = 0.036; η2 = 0.247; and π = 0.748], but not between positive and neutral neither between negative and positive [*F*(1,23) = 1.01; *p >* 0.1; η2 = 0.042; and π = 0.161 and *F*(1,23) = 2.05; *p >* 0.1; η2 = 0.082; and π = 0.278, respectively]. This matches visual inspection of the waves, in which the P600 appeared for the three valences, being larger for the neutral condition, followed by the positive one, the smallest being for the negative one, then reversing the results of the LAN (Figure 3). Preceding task did not yield significant effects, neither as main effect nor in interaction with the other factors [all *F*s < 2.04, *p* always >0.1].

## Discussion

The major issue addressed in this study has been to investigate long-term emotional valence modulations induced by words on subsequent morphosyntactic processing when arousal levels are equated. Whether these effects persist across different degrees of processing of those affective words (by means of performing two different tasks at that moment) was also assessed. Although significant differences were found between ES and RA preceding tasks in SC measurement, morphosyntactic processing was not affected by the preceding task type. In contrast, even though SC differences were not observed for valence, emotional valence modulations were observed on the main ERP components related to morphosyntactic processing occurring around 2 s later. Both negative and positive words seemed to enhance the amplitude of the LAN and to reduce the P600 component. Negative valence significantly differed from neutral condition both in the LAN and in the P600 ERP components. There were, however, no significant differences between negative and positive neither between positive and neutral conditions, neither in the LAN nor in the P600 for this comparison. Visual inspection, however, rather suggests an intermediate position of the positive condition between the negative and the neutral ones, although this depiction should be viewed with caution.

Similarly to the present experiment, larger LAN amplitudes for negative and positive conditions in comparison to the neutral one were observed in Jiménez-Ortega et al. (2012). As in the present study, the emotional information preceded the morphosyntactic judgment task (i.e., long term effects). Specifically, Jiménez-Ortega et al. (2012) presented emotional paragraphs before neutral sentences containing the morphosyntactic anomalies (see Introduction section). The present experiment would shed light on those previous results. First, in Jiménez-Ortega et al. (2012) experiment it was not possible to disentangle whether the effects on the LAN were due either to valence or to arousal, since arousal for both negative and positive paragraphs was always higher in comparison to the neutral ones. Current findings, in which arousal is equated in the three emotional conditions, i.e., including the neutral one, indicate that most probably the effects on the LAN in Jiménez-Ortega et al. (2012) were mainly due to emotional valence. Since in Vissers et al. (2010) and Verhees et al. (2015) no neutral condition was included, it is difficult to directly extrapolate the present data to understand their finding of a reduced P600 to negative (sad) mood as compared to positive mood. Although our data suggest that the P600 in the negative condition was more reduced that in the positive one, which in turn was so in comparison to the neutral one, although positive and negative conditions did not differ statistically. Accordingly, it is possible that our pattern of results (less P600 amplitude for negative as compared to positive conditions) parallels those by Vissers et al. (2010) and Verhees et al. (2015), but this was not supported because of statistical power problems.

Our results seem to reflect a pattern in which, overall, emotional valences increase LAN amplitude while decreasing P600. This pattern relates to previous reports in which, e.g., individuals with better working memory capacities normally show a larger LAN/reduced P600 in comparison to bad comprehenders or individuals with lower working memory capacity (King and Kutas, 1995; Vos et al., 2013; Tanner and Van Hell, 2014). Applying this framework to our data, it is plausible that the present pattern may be reflecting a more efficient processing of morphosyntactic information when emotional information precedes the sentence in which a morphosyntactic violation occurs, as compared to neutral conditions. Overall, accordingly, emotional valence seems to boost the processing of subsequent morphosyntactic information.

It is interesting to note that in Espuny et al. (2018), short-term effects of the emotional valence, in terms of customary ERP modulations to emotional content (EPN and LPC) only emerged for the negative words, while in the present study, exploring long-term effects of valence, both positive and negative words impacted morphosyntactic processing similarly. This is true even regardless of the manner of processing the word, i.e., whether it was normally (RA task) or hardly read, that is, with content access inhibition and conflict, among other factors (ES task). Overall, the finding would reveal that emotional information is powerful enough as to affect syntactic processing even when processed in impoverished circumstances, in line with previous studies with subliminal presentations of emotional material within the sentence (Jiménez-Ortega et al., 2017).

Valence seems to denote that there is relevant information for the subject (negative and positive valence versus neutral words). As arousal effects can be entirely disregarded in the present findings, only valence information can uphold. To the extent that the effect was independent common for either type of valence, a general effect must be accounting for our data. It is possible that the semantic content associated to emotional valences triggers an overall strategy by which enhanced processing follows, and this would convey the increase of cognitive resources devoted to process the subsequently appearing sentence. This would in turn result in enhanced LAN/reduced P600, resembling the pattern reported for individuals with larger working memory capacities, as discussed above.

Indeed, emotional information is part of the semantic content of a word (Binder and Desai, 2011). Therefore, our results contradict theories of syntactic processing as an encapsulated module, assumed to be unaffected by other processes such as semantic information (Hauser et al., 2002; Friederici, 2004). The present findings are rather in line with interactive models (see, e.g., Pulvermüller et al., 2001), according to which semantic and syntactic information interact from early stages of language processing. Our findings provide evidence favoring that neural networks supporting emotions are wide and intermixed with networks underlying cognitive processing, as proposed by Pessoa (2008, 2013).

Our results might also be in line with findings in which positive and negative emotions interact differently with attention (Srinivasan and Gupta, 2010; Gupta and Srinivasan, 2015; Gupta et al., 2016, see also: Maidhof and Koelsch, 2011). In this regard, positive emotions seem to require less attentional resources than negative ones (Srivastava and Srinivasan, 2010; Gupta and Srinivasan, 2015; Gupta et al., 2016), the latter also requiring more focused attention (Srinivasan and Gupta, 2010). This would be in consonance with our seemingly differential results between negative and positive valences on LAN and P600 components, possibly reflecting that the negative conditions enhanced subsequent sentence processing in a higher degree than the positive ones. However, as the differences between our positive and negative conditions were not supported statistically, probably due to statistical power problems, the interpretation should be taken with caution.

## Conclusion

In conclusion, our data suggest that the emotional valence of a word can affect morphosyntactic processing occurring about 2 s later, and that this occurs even when the emotion-laden word has been poorly processed. The result contributes not only to better acknowledge the interactions between emotional-semantic and syntactic processes, but also to recognize that the emotional words that appear in a text, a discourse or in conversations may convey effects on the morphosyntactic processing of sentences appearing far apart.

## Author Contributions

JE, LJ-O, and MM-L designed the study, interpreted the data, discussed the results, and wrote the manuscript. JE and LJ-O collected the data and prepared the data for analysis. All authors revised the manuscript.

## Conflict of Interest Statement

The authors declare that the research was conducted in the absence of any commercial or financial relationships that could be construed as a potential conflict of interest.

## References

[B1] Ashby F. G. Isen A. M. Turken A. U. (1999). A neuropsychological theory of positive affect and its influence on cognition. *Psychol. Rev.* 106 529–550. 10.1037/0033-295X.106.3.529 10467897

[B2] Binder J. R. Desai R. H. (2011). The neurobiology of semantic memory. *Trends Cogn. Sci.* 15 527–536. 10.1016/j.tics.2011.10.001 22001867 PMC3350748

[B3] Bohn K. Knaus J. Wiese R. Domahs U. (2013). Neuropsychologia The influence of rhythmic ( ir ) regularities on speech processing: evidence from an ERP study on German phrases. *Neuropsychologia* 51 760–771. 10.1016/j.neuropsychologia.2013.01.006 23333869

[B4] Bradley M. M. Lang P. J. (1999). *Affective Norms for English Words ( ANEW ): Instruction Manual and Affective Ratings*. Gainesville, FL: University of Florida, Center for Research in Psychophysiology.

[B5] Coulson S. King J. W. Kutas M. (1998). Expect the unexpected: event-related brain response to morphosyntactic violations. *Lang. Cogn. Process.* 13 21–58. 10.1080/016909698386582

[B6] Dresler T. Mériau K. Heekeren H. R. Van Der Meer E. (2009). Emotional stroop task: effect of word arousal and subject anxiety on emotional interference. *Psychol. Res.* 73 364–371. 10.1007/s00426-008-0154-6 18636272

[B7] Espuny J. Jiménez-Ortega L. Casado P. Fondevila S. Muñoz F. Hernández-Gutiérrez D. (2018). Event-related brain potential correlates of words’ emotional valence irrespective of arousal and type of task. *Neurosci. Lett.* 670 83–88. 10.1016/j.neulet.2018.01.050 29391218

[B8] Friederici A. D. (2004). “The neural basis of syntactic processes,” in *The Cognitive Neurosciences*, 3rd Edn, ed. Gazzaniga M. S. (Cambridge, MA: The MIT Press), 805–818.

[B9] Friederici A. D. Hahne A. Saddy D. (2002). Distinct neurophysiological patterns reflecting aspects of syntactic complexity and syntactic repair. *J. Psycholinguist. Res.* 31 45–63. 10.1023/A:1014376204525 11924839

[B10] Gupta R. Hur Y. J. Lavie N. (2016). Distracted by pleasure: effects of positive versus negative valence on emotional capture under load. *Emotion* 16 328–337. 10.1037/emo0000112 26479771

[B11] Gupta R. Raymond J. E. (2012). Emotional distraction unbalances visual processing. *Psychon. Bull. Rev.* 19 184–189. 10.3758/s13423-011-0210-x 22227946

[B12] Gupta R. Raymond J. E. Vuilleumier P. (2018). Priming by motivationally salient distractors produces hemispheric asymmetries in visual processing. *Psychol. Res.* 0 1–10. 10.1007/s00426-018-1028-1 29797045

[B13] Gupta R. Srinivasan N. (2015). Only irrelevant sad but not happy faces are inhibited under high perceptual load. *Cogn. Emot.* 29 747–754. 10.1080/02699931.2014.933735 24998248

[B14] Hagoort P. (2003). Interplay between syntax and semantics during sentence comprehension: ERP effects of combining syntactic and semantic violations. *J. Cogn. Neurosci.* 15 883–899. 10.1162/089892903322370807 14511541

[B15] Hahne A. Friederici A. D. (1999). Electrophysiological evidence for two steps in syntactic analysis. Early automatic and late controlled processes. *J. Cogn. Neurosci.* 11 194–205. 10.1162/089892999563328 10198134

[B16] Hauser M. D. Chomsky N. Fitch W. T. (2002). The faculty of language: what is it, who has it, and how did it evolve? *Science* 298 1569–1579. 10.1126/science.298.5598.1569 12446899

[B17] Jiménez-Ortega L. Espuny J. de Tejada P. H. Vargas-Rivero C. Martín-Loeches M. (2017). Subliminal emotional words impact syntactic processing: evidence from performance and event-related brain potentials. *Front. Hum. Neurosci.* 11:192. 10.3389/fnhum.2017.00192 28487640 PMC5404140

[B18] Jiménez-Ortega L. García-Milla M. Fondevila S. Casado P. Hernández-Gutiérrez D. Martín-Loeches M. (2014). Automaticity of higher cognitive functions: neurophysiological evidence for unconscious syntactic processing of masked words. *Biol. Psychol.* 103 83–91. 10.1016/j.biopsycho.2014.08.011 25161085

[B19] Jiménez-Ortega L. Martín-Loeches M. Casado P. Sel A. Fondevila S. de Tejada P. H. (2012). How the emotional content of discourse affects language comprehension. *PLoS One* 7:33718. 10.1371/journal.pone.0033718 22479432 PMC3315581

[B20] King J. W. Kutas M. (1995). Who did what and when? Using word- and clause-level ERPs to monitor working memory usage in reading. *J. Cogn. Neurosci.* 7 376–395. 10.1162/jocn.1995.7.3.376 23961867

[B21] Lang P. J. Bradley M. M. Cuthbert B. N. (1998). Emotion, motivation, and anxiety: brain mechanisms and psychophysiology. *Biol. Psychiatry* 44 1248–1263. 10.1016/S0006-3223(98)00275-3 9861468

[B22] Magne C. Jordan D. K. Gordon R. L. (2016). Speech rhythm sensitivity and musical aptitude: ERPs and individual differences. *Brain Lang.* 15 13–19. 10.1016/j.bandl.2016.01.001 26828758

[B23] Maidhof C. Koelsch S. (2011). Effects of selective attention on syntax processing in music and language. *J. Cogn. Neurosci.* 23 2252–2267. 10.1162/jocn.2010.21542 20617885

[B24] Martín-Loeches M. Fernández-Hernández A. Schacht A. Sommer W. Casado P. Jiménez-Ortega L. (2012). The influence of emotional words on sentence processing: electrophysiological and behavioral evidence. *Neuropsychologia* 50 3262–3272. 10.1016/j.neuropsychologia.2012.09.010 22982604

[B25] Martín-Loeches M. Muñoz F. Casado P. Melcón A. Fernández-Frías C. (2005). Are the anterior negativities to grammatical violations indexing working memory? *Psychophysiology* 42 508–519. 10.1111/j.1469-8986.2005.00308.x 16176373

[B26] Martín-Loeches M. Schacht A. Casado P. Hohlfeld A. Abdel Rahman R. Sommer W. (2009). Rules and heuristics during sentence comprehension: evidence from a dual-task brain potential study. *J. Cogn. Neurosci.* 21 1365–1379. 10.1162/jocn.2009.21106 18752393

[B27] Mitchell R. L. C. Phillips L. H. (2007). The psychological, neurochemical and functional neuroanatomical mediators of the effects of positive and negative mood on executive functions. *Neuropsychologia* 45 617–629. 10.1016/j.neuropsychologia.2006.06.030 16962146

[B28] Oldfield R. C. (1971). The assessment and analysis of handedness: the Edinburgh inventory. *Neuropsychologia* 9 97–113. 10.1016/0028-3932(71)90067-4 5146491

[B29] Pessoa L. (2008). On the relationship between emotion and cognition. *Nat. Rev. Neurosci.* 9 148–158. 10.1038/nrn2317 18209732

[B30] Pessoa L. (2013). *The Cognitive-Emotional Brain: From Interactions to Integration.* Cambridge, MA: MIT Press, 10.1073/pnas.0703993104

[B31] Pulvermüller F. Härle M. Hummel F. (2001). Walking or talking? Behavioral and neurophysiological correlates of action verb processing. *Brain Lang.* 78 143–168. 10.1006/brln.2000.2390 11500067

[B32] Redondo J. Fraga I. Padrón I. Comesaña M. (2007). The Spanish adaptation of anew (Affective Norms for English Words). *Behav. Res. Methods* 39 600–605. 10.3758/BF03193031 17958173

[B33] Sassenhagen J. Bornkessel-Schlesewsky I. (2015). The P600 as a correlate of ventral attention network reorientation. *Cortex* 66 3–20. 10.1016/j.cortex.2014.12.019 25791606

[B34] Spielberger C. (1983). *Manual for the State-Trait Anxiety Inventory (STAI)*. Washington, DC: Consulting Psychologists Press, 4–26.

[B35] Srinivasan N. Gupta R. (2010). Emotion-attention interactions in recognition memory for distractor faces. *Emotion* 10 207–215. 10.1037/a0018487 20364896

[B36] Srinivasan N. Gupta R. (2011). Rapid communication: global–local processing affects recognition of distractor emotional faces. *Q. J. Exp. Psychol.* 64 425–433. 10.1080/17470218.2011.552981 21347993

[B37] Srinivasan N. Hanif A. (2010). Global-happy and local-sad: perceptual processing affects emotion identification. *Cogn. Emot.* 24 1062–1069. 10.1080/02699930903101103

[B38] Srivastava P. Srinivasan N. (2010). Time course of visual attention with emotional faces. *Attent. Percept. Psychophys.* 72 369–377. 10.3758/APP 20139452

[B39] Tanner D. Van Hell J. G. (2014). ERPs reveal individual differences in morphosyntactic processing. *Neuropsychologia* 56 289–301. 10.1016/j.neuropsychologia.2014.02.002 24530237

[B40] Teixeira-Silva F. Prado G. B. Ribeiro L. C. G. Leite J. R. (2004). The anxiogenic video-recorded stroop color-word test: psychological and physiological alterations and effects of diazepam. *Physiol. Behav.* 82 215–230. 10.1016/j.physbeh.2004.03.031 15276783

[B41] Verhees M. W. F. T. Chwilla D. J. Tromp J. Vissers C. T. W. M. (2015). Contributions of emotional state and attention to the processing of syntactic agreement errors: evidence from P600. *Front. Psychol.* 6:388. 10.3389/fpsyg.2015.00388 25914660 PMC4391228

[B42] Vissers C. T. W. M. Virgillito D. Fitzgerald D. A. Speckens A. E. M. Tendolkar I. van Oostrom I. (2010). The influence of mood on the processing of syntactic anomalies: evidence from P600. *Neuropsychologia* 48 3521–3531. 10.1016/j.neuropsychologia.2010.08.001 20696180

[B43] Vos S. H. Gunter T. C. Kolk H. H. J. Mulder G. (2013). Working memory constraints on syntactic processing: an electrophysiological investigation. *Psychophysiology* 38 41–63. 11321620

